# Long ignored but making a comeback: a worldwide epidemiological evolution of human brucellosis

**DOI:** 10.1080/22221751.2023.2290839

**Published:** 2023-12-01

**Authors:** Zhiguo Liu, Liping Gao, Miao Wang, Min Yuan, Zhenjun Li

**Affiliations:** a National Key Laboratory of Intelligent Tracking and Forecasting for Infectious Diseases, National Institute for Communicable Disease Control and Prevention, Chinese Center for Disease Control and Prevention, Beijing, People’s Republic of China; b National Key Laboratory of Intelligent Tracking and Forecasting for Infectious Diseases, National Institute for Viral Disease Control and Prevention, Chinese Center for Disease Control and Prevention, Beijing, People’s Republic of China; c Ulanqab Center for Disease Control and Prevention, Jining (Inner Mongolia), People’s Republic of China; d Chinese Center for Disease Control and Prevention, Beijing, People’s Republic of China

**Keywords:** Human brucellosis, cases, incidence rate, seropositive, epidemiological evolution

## Abstract

Brucellosis is a commonly neglected zoonosis that remains a serious global public health concern. The epidemiological evolution of human brucellosis has considerably changed over the past few decades, and epidemic geography is continuously expanding. Human brucellosis is emerging and re-emerging, and is imported from areas where it is endemic due to travel, immigration, and international trade. The disease continues to be prevalent in Asia and Africa, including West Asia, Central Asia, North Africa, and East Africa, with the highest incidence in Syria, Kyrgyzstan, Mongolia, Iran, Algeria, and Kenya. Re-emerging cases are frequently recorded in places where brucellosis has been controlled, such as Bosnia, Herzegovina, Azerbaijan, and the USA. In countries with a high disease burden, disease control and eradication have been extremely difficult because of livestock farming being the only source of livelihood, unique religious beliefs regarding animals, nomadic lifestyle, and low socioeconomic levels. Interventions focused on protecting livestock keepers are needed, particularly for those assisting with goat and sheep births and the consumption of raw dairy products. Notably, in most countries with a high disease burden, each period of several years with a low incidence rate was followed by a subsequent increase in cases, highlighting the necessity of continuous investment and surveillance. In addition, advocacy for the inclusion of brucellosis as a globally mandated reported disease, strict restrictions on animal movement, mandated consumption of pasteurized milk, and health education are needed. This study will help form an evidence-based strategy for international organizations to curb the future spread of brucellosis.

## Introduction

The World Health Organization currently classifies brucellosis as one of the top neglected zoonoses worldwide. Despite its high burden in several developing countries, the disease is often neglected. Brucellosis has been an endemic disease worldwide since the discovery of *Brucella melitensis* (*B. melitensis*) by David Bruce on Malta Island in 1887 [1]. Although at least 170 countries have reported human brucellosis cases, brucellosis remains the most common but often neglected disease. Global commerce and trade, international travel, animal-to-human interactions, destruction, and destruction of ecological systems contribute to the ongoing threat of emerging and re-emerging infectious diseases. This poses a risk to public health and socioeconomic advances, and allows for the emergence of new and unrecognized disease-causing microbial agents [2].

Many industrialized countries have eradicated *B. abortus* brucellosis in cattle; however, in many livestock farming countries *B. melitensis* and *B. suis* have emerged as causative agents of brucellosis among goats, sheep, cattle, and other livestock, leading to numerous human cases [3]. A high incidence rate of brucellosis in humans has been observed in many regions, including South America, Africa, the Middle East, and many parts of Asia [4]. In addition, global public health concerns regarding brucellosis infection are increasing, encompassing concerns with laboratories, vaccines, and new infections arising from consumption of raw dairy products [5]. Understanding the epidemiological trend of brucellosis is imperative for fulfilling the “one health” concept. Therefore, this study aimed to summarize the latest global epidemiological map of brucellosis and shed light on the epidemic trends. This will aid in formulating strengthened surveillance and control strategies for brucellosis based on a single health approach.

## Epidemiological profile of brucellosis globally

**Figure 1.**
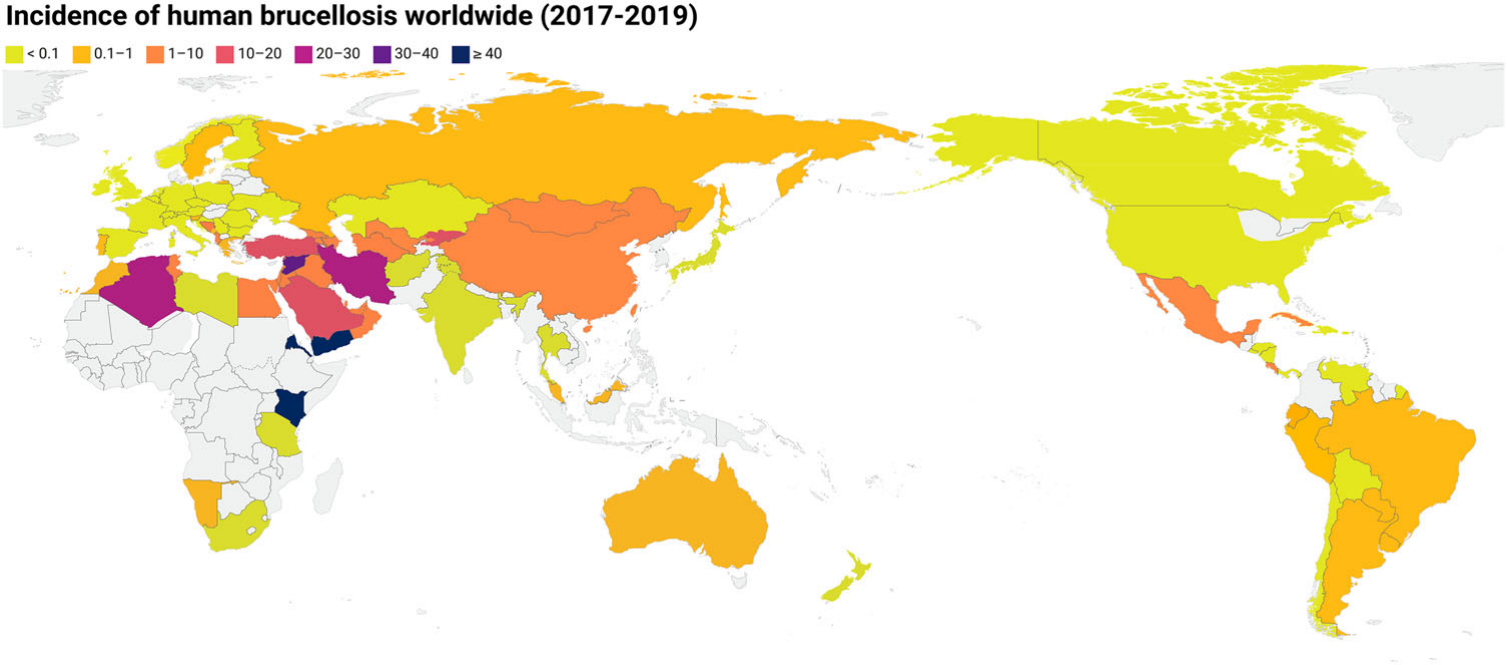
The map of the global brucellosis epidemiological profile (the incidence rates of human brucellosis from public open-source from OIE-WAHIS databases, Table S1). Note: Incidence rate (/100,000) from 2017 to 2019 was used to depict the map of global human brucellosis, the gray indicates areas with no data. The map from Standard Map Services Website (http://bzdt.ch.mnr.gov.cn/), and approval number of map: GS (2016) 1666.

Human brucellosis has changed significantly over the past decade (Table 1) compared to its status in 2006 [4]. The epidemic territory has significantly expanded, from 53 countries to at least 97 countries, including 18 additional countries in Europe, 12 in the Americas, nine in Asia, and four in Africa (Table 1). Certainly, the increased number of countries is partially related to improvements in diagnosis and surveillance. The worldwide incidence of human brucellosis is depicted in Figure 1 (data from OIE-WAHIS). The highest incidence of brucellosis has been reported in the Eastern Mediterranean region, including Syria, Turkey, Iraq, Saudi Arabia, Oman, and Algeria. The incidence of brucellosis ranges from 0.029/100,000 to 200.41/100,000 in this area; however, considering the poor surveillance systems among the countries in the region, the actual incidence is estimated to be 20–25 times greater.

**Table 1.** Comparative analysis of global human brucellosis in 2006 and 2019.

Continent	Areas	Countries	Incidence (cases/100,000)	
			2006	2019 (2017-2019)
Africa	North Africa	Egypt	0.295	4.154 (2014)
		Algeria	8.43	27.05 (2018)
		Morocco	–	0.239
		Tunisia	3.54	8.621 (2018)
		Libya	–	0.045 (2018)
	West Africa	Cabo Verde	–	0.186 (2017)
		Mali	0.2	–
	East Africa	Kenya	–	293.103
		Eritrea	0.548	44.109 (2018)
		Uganda	0.09	–
	South Africa	Namibia	0.49	0.160
Americas	North America	United States of America	0.04	0.043 (2018)
		Canada	0.012	0.025 (2016)
		Mexico	2.87	1.304
		Cuba	–	0.132 (2018)
		Costa Rica	–	0.82 (2018)
		Honduras	–	0.021 (2017)
		Panama	1.01	0.024 (2017)
		Nicaragua	–	0.031
		El Salvador	–	0.016
		Dominican Republic (the)	–	0.0094 (2018)
		Guatemala	1.57	0.02 (2012)
	South America	Colombia	0.185	0.053 (2015)
		Venezuela (Bolivarian Republic of)	–	0.021 (2018)
		Ecuador	–	0.259
		Peru	3.49	0.344 (2018)
		Paraguay	–	0.156
		Argentina	0.84	0.532
		Brazil	–	0.112
		Uruguay	–	0.116
		Chile	0.06	0.016 (2018)
		Bolivia (Plurinational State of)	–	0.009 (2018)
Asia	East Asia	China	1.45	3.154
		Mongolia	60.59	3.101
		Japan	–	0.005 (2018)
		Korea (the Democratic People's Republic of)	0.1	–
		Korea (the Republic of)	–	0.010 (2015)
	West Asia	Afghanistan	0.38	0.055
		Iran (Islamic Republic of)	23.86	20.332 (2018)
		Turkey	26.22	12.279
		Syrian Arab Republic	160.34	36.643
		Lebanon	4.95	3.295 (2008)
		Palestine	–	15.32 (2018)
		Israel	0.92	3.044
		Jordan	2.34	3.01 (2018)
		Iraq	27.84	2.892
		Kuwait	3.39	9.817
		Saudi Arabia	21.44	14.175 (2017)
		Yemen	–	157.597 (2018)
		Oman	3.56	6.336 (2018)
		United Arab Emirates (the)	4.1	1.013
		Qatar	–	4.343
		Georgia	2.76	5.054
		Armenia	3.13	8.317
		Azerbaijan	5.26	5.586
	Central Asia	Turkmenistan	5.15	7.824 (2016)
		Uzbekistan	1.8	2.358 (2018)
		Kyrgyzstan	36.22	12.447 (2018)
		Tajikistan	21.19	9.71 (2015)
		Kazakhstan	11.58	0.038
	Souther Asia	India	–	0.002 (2017)
		Bhutan	–	0.340
	Southeast Asia	Thailand	–	0.032
		Malaysia	–	0.129
	North Europe	Denmark	0.07	–
		Ireland	0.13	0.042 (2017)
		Sweden	0.03	0.137
		Norway	0.07	0.075
		Finland	–	0.018 (2017)
	West Europe	France	0.05	0.051
		Luxembourg	–	0.174 (2016)
		Belgium	–	0.260
		Northern Ireland (the)	0.03	0.036
		Netherlands (the)	0.05	0.040
	Central Europe	Germany	0.03	0.045
		Austria	–	0.068
		Switzerland	0.15	0.082
		Poland	–	0.005
		Czechia	–	0.037
		Slovakia	–	0.018
	East Europe	Ukraine	–	0.007 (2017)
		Russian Federation (the)	0.41	0.275
		Estonia	–	0.075
		Lithuania	–	0.068 (2013)
	South Europe	Greece	2.09	0.606
		Italy	0.9	0.082
		Spain	1.51	0.043
		Portugal	1.39	0.321
		Romania	–	0.005
		Bulgaria	–	0.028 (2018)
		Serbia	–	0.029
		Montenegro	0.84	0.321 (2018)
		Slovenia	–	0.288
		Croatia	–	0.074
		Bosnia and Herzegovina	2.08	6.241
		North Macedonia (Rep. of)	14.8	0.720
		Albania	6.36	1.221 (2018)
		Andorra	–	1.3 (2016)
Oceania		Australia	0.09	0.108 (2018)
		New Zealand	–	0.060

Note: the year in brackets refers to the incidence rate in the year; “–”: indicates that no data.

**Figure 2.**
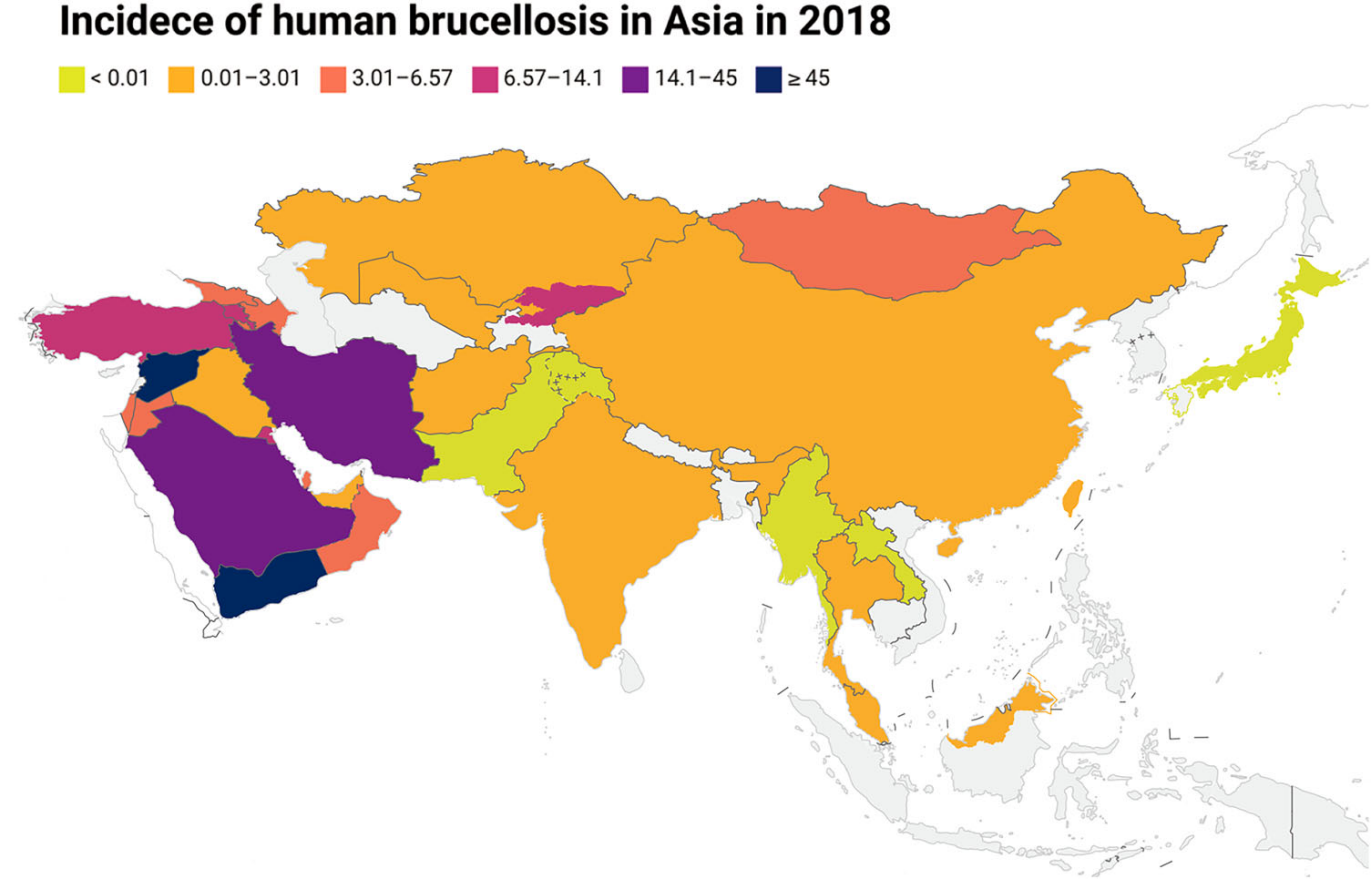
The map of the brucellosis epidemiological profile in Asian countries in 2018. Note: Incidence rate (/100,000) of human brucellosis from public open-source from OIE-WAHIS databases, the gray indicates areas with no data. The map from Standard Map Services Website (http://bzdt.ch.mnr.gov.cn/), and approval number of map: GS (2016) 1666.

Notably, there was a significant increase in cases in some Asian countries; however, the annual incidence in Europe and America showed a declining trend (Table 1). Brucellosis remains the most serious public health and socioeconomic concern in Asia, with new cases being consistently reported. (Figure 2). Currently, European countries have a significantly declining trend in cases; however, three countries have a high incidence in Europe, including Bosnia and Herzegovina, North Macedonia, and Greece (Table 1). In America, some countries are still hotspot areas. Human brucellosis is continuously reported in endemic countries, including Mexico, Peru, Argentina, and Brazil. Oceania is a human brucellosis-free continent, with only sporadic cases reported in Australia and New Zealand. The reported and surveillance data on human brucellosis are still insufficient in Africa. Epidemiological surveillance in African countries is a great challenge due to weak public health systems and low socioeconomic development.

## Asia

Asia has the highest human brucellosis burden among continents, especially in Eastern, Central, and Western Asian countries, which poses a serious public health problem (Figure 2). Persistent traditional agricultural practices and lifestyles, and consumption of fresh dairy products, such as raw milk, contribute to this high prevalence. Although the majority of countries have seen a decreasing trend in human brucellosis from 2005 to 2019, the incidence rate is still high compared with countries on other continents.

In Eastern Asia, the highest disease burden is observed in China and Mongolia, with the incidence gradually increasing and geography continuously expanding. In China, the incidence rate increased from 0.0281/100,000 in 1993 to 5.0553/100,000 in 2021 (Table 2). All 31 mainland provinces have reported human cases, and affected regions have expanded to include southern regions. Although more than 95% of the disease burden is in northern areas, such as Inner Mongolia, the incidence (cases) in southern regions is stable at lower levels (Table 2). *B. melitensis* strains dominate as the circulating species in all regions and drive brucellosis transmission from the north to the south [6]. Therefore, a priority measure is enhancing surveillance and intervention of brucellosis in northern provinces. In Mongolia, although there was a significant decrease in the incidence rate of human brucellosis from 16.23/100,000 in 2007 to 3.10/100,000 in 2019, the human brucellosis seroprevalence among rural people in Mongolia is high, with one survey showing that there was a 15-fold underreporting of human brucellosis [7]. Some sporadic cases were reported in Japan, South Korea, Hong Kong, and Taiwan. Human brucellosis rarely occurs in Japan, and only a few *B. canis* infection cases have been reported [8]. In South Korea, the first case of human brucellosis was reported in 2002, and cases of human infection continue to occur. The hotspots of human brucellosis were clustered in the southeast regions of Korea. The risk of human brucellosis increases in rural regions, with the highest risk from bovine brucellosis [9]. The first case of human brucellosis caused by *B. melitensis* [10] and a foodborne outbreak of human brucellosis (n = 5) in South Korea was reported between 2012 and 2013 [11]. In Hong Kong, six patients were diagnosed with brucellosis in 2010. All patients were diagnosed by positive blood culture of *B. melitensis;* importantly*,* diagnosis was suspected for only one patient at presentation, implying that human brucellosis may be underdiagnosed [12]. In Taiwan, the first domestic case of human brucellosis was reported in a graduate student in 1978 who acquired the infection during laboratory work. Subsequently, 16 human brucellosis cases were reported in 1979, nine of whom were laboratory personnel. Subsequently, four cases were recorded in 2012, and all four patients had a travel history to countries with brucellosis and a relevant animal or food history [13]. A high risk of imported and laboratory infection of human brucellosis still exists, and surveillance and proper protection are recommended.

**Table 2.** Comparative analysis of human brucellosis in 1993 and 2021 in China.

Areas		1993		2021	
		No. of Cases	Incidence (/100,000)	No. of Cases	Incidence (/100,000)
Northern	Xizang (Tibet)	171	7.5307	43	1.1787
	Beijing	–	–	83	0.3791
	Tianjin	–	–	233	1.6804
	Qinghai	–	–	756	12.7618
	Jilin	1	0.004	1265	5.2548
	Shaanxi	1	0.003	1402	3.5468
	Shandong	14	0.0163	3323	3.273
	Heilongjiang	1	0.0028	4048	12.7095
	Gansu	1	0.0043	4562	18.2335
	Hebei	37	0.0589	4664	6.2512
	Xinjiang	1	0.0064	4780	18.4896
	Shanxi	79	0.269	4823	13.8133
	Henan	5	0.0056	4888	4.9192
	Ningxia	1	0.0206	4943	68.6277
	Liaoning	1	0.0025	5449	12.7937
	Inner Mongolia	2	0.0091	21313	88.6228
Southern	Shanghai	–	–	5	0.0201
	Hainan	–	–	20	0.1984
	Chongqing	–	–	70	0.2184
	Guizhou	–	–	89	0.2308
	Jiangxi	–	–	103	0.2279
	Hubei	–	–	104	0.1801
	Zhejiang	–	–	178	0.2757
	Fujian	–	–	182	0.4381
	Sichuan	5	0.0046	199	0.2378
	Guangxi	2	0.0046	224	0.4469
	Hunan	–	–	239	0.3597
	Jiangsu	–	–	285	0.3363
	Anhui	–	–	340	0.5571
	Guangdong	4	0.0061	455	0.3611
	Yunnan	–	–	699	1.4806

Note: “–” : No reported case, the data from previously published study (DOI: 10.46234/ccdcw2023.004).

The disease burden of human brucellosis in Southeast Asian countries, such as Malaysia and Vietnam, is relatively small, and only sporadic cases have been reported in Thailand. In Malaysia, despite human brucellosis having low prevalence rates, the disease has serious public health implications. In 2011, an outbreak of more than 30 cases occurred in Penang. From 2014 to 2019, 5.8% (74/1,281) of suspected cases showed seropositivity for human brucellosis. The central region had the highest seropositive cases of human brucellosis, and consumption of unpasteurized milk was significantly associated with these cases [14]. In Vietnam, brucellosis is not a commonly reported cause of febrile disease, and no cases have been reported since three cases of *B. abortus* in 1962. Ten human *B. melitensis* infection cases were reported in southern Vietnam from 2016 to 2017, and these *B. melitensis* were clustered with organisms originating from Southern Europe, the Middle East, and China [15]. Brucellosis was previously a re-emerging disease in Thailand, with only eight human brucellosis cases reported, all caused by *B. melitensis* before 2014 [16]. In 2019, a survey showed that 3.7% of pregnant women had seropositive results for anti-*Brucella abortus* IgG in Thailand, and rearing goats at home or consuming raw goat products was the main transmission route [17]. Therefore, a persistent surveillance and control plan in these low-incidence regions is vital for evaluating the epidemic trend of the disease.

**Figure 3.**
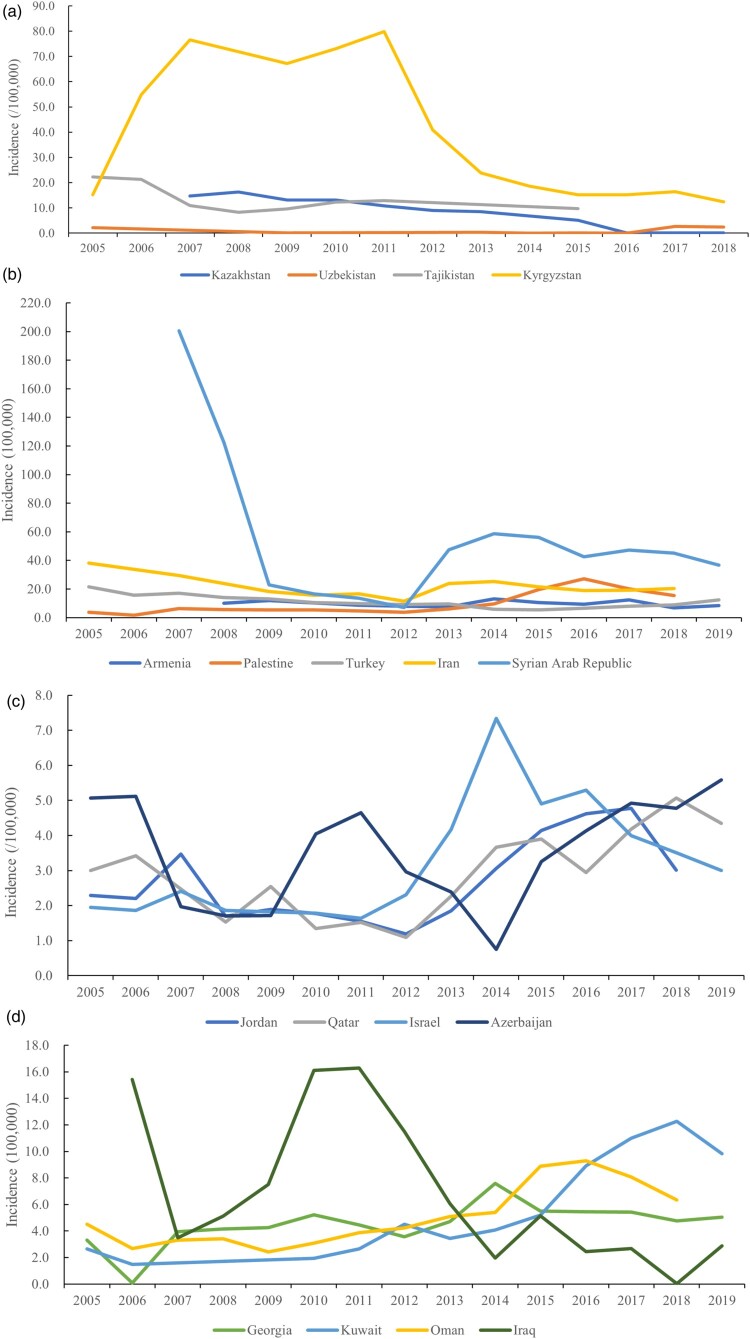
The incidence rate (/100,000) profile in Asian countries (a–d) with a high disease burden, from 2005 to 2019. Note: the number of cases and the incidence rates of human brucellosis are from public open-source from OIE-WAHIS databases.

**Figure 4.**
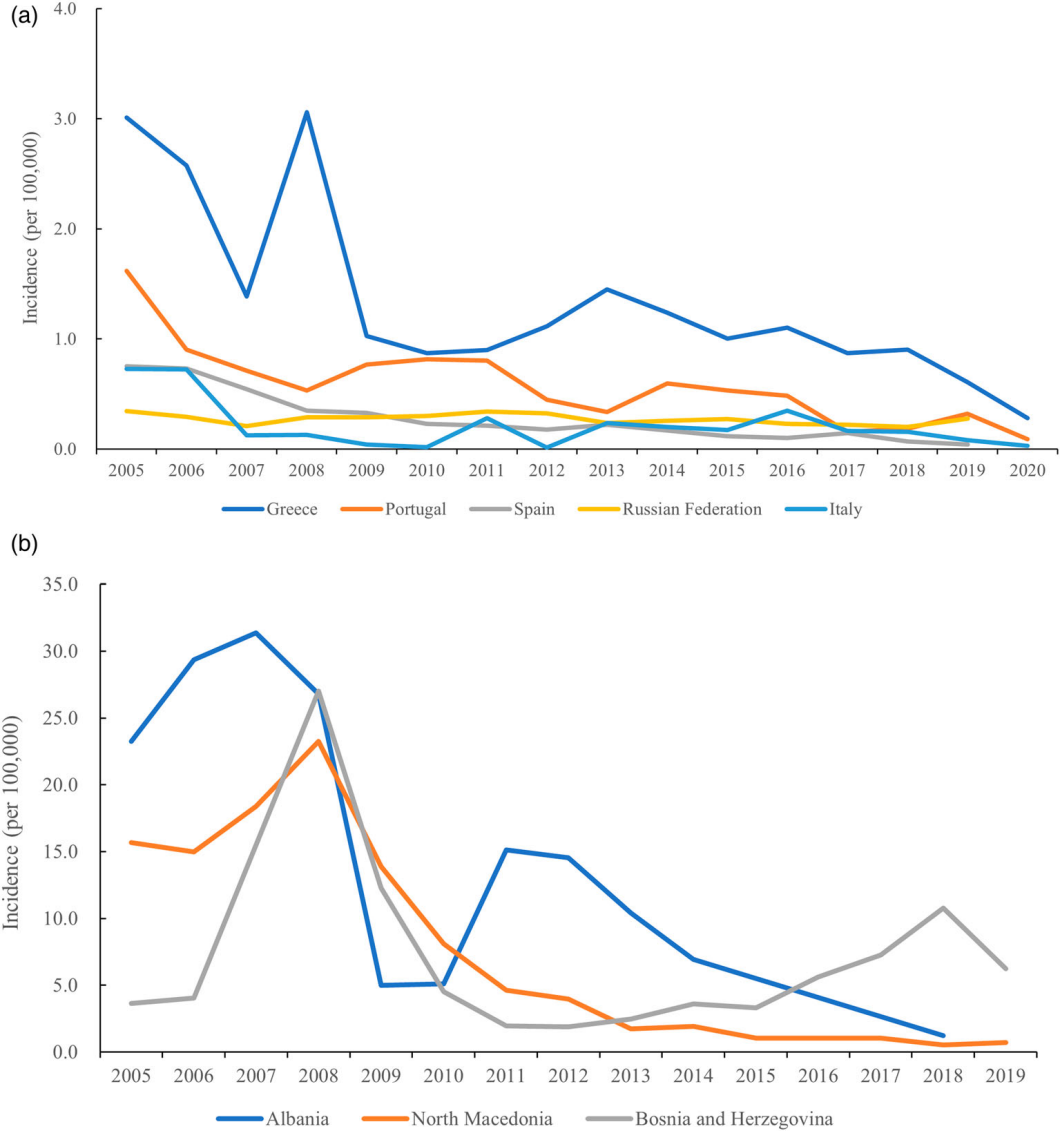
The incidence rate (/100,000) profile in European countries (a and b) with a high disease burden, from 2005 to 2019. Note: the number of cases and the incidence rates of human brucellosis are from public open-source from OIE-WAHIS databases.

Despite a decrease in incidence rate, Central Asia, including Kazakhstan, Kyrgyzstan, and Tajikistan, remains one of the highest-incidence areas for human brucellosis in the world (Figure 4a). Kazakhstan has one of the highest incidences of human brucellosis worldwide. The brucellosis epidemic most likely began before the collapse of the USSR. High livestock densities as well as changes to the livestock systems may have played an important role. In Kazakhstan, the incidence of human brucellosis declined from 14.71/100,000 in 2007 to 0.038/100,000 in 2019 (Table S1). This was achieved by implementing an obligatory vaccination program with varying levels of coverage from 2001 to 2006 [18]. Human Brucellosis remains a challenging health problem in Kyrgyzstan. The incidence was low during 1950–1990, increased significantly from 1991 to 2011, and declined from 2012 to 2020 [19]. The incidence rate fluctuated increasing from 15.24/100,000 in 2005 to a peak of 79.82/100,000, and then decreased to 12.45/ 100,000 in 2018 (Figure 3a). In Uzbekistan, the incidence rate was 2.12/100,000 in 2005, declined to 0.04/100,000 in 2016, and then substantially increased to 2.39/100,000 in 2018. Although the disease is a public health threat in Uzbekistan, the disease surveillance is challenging due to limited laboratory capacity and surveillance for the disease is hospital-based [20]. In Tajikistan, brucellosis has been widespread in humans, although the incidence rate decreased from 22.27/100,000 in 2005 to 9.71 /100,000 in 2015. Although consumption and trading of unpasteurized dairy products are common in Tajikistan, this situation might constitute a serious population health concern [21].

In southern Asia, human brucellosis is endemic, especially in Afghanistan, Nepal, and India. Brucellosis is known to be endemic in humans throughout Afghanistan, although a sharp decline was observed from 2007 (11.13/100,000) to 2019 (0.06/100,000). The latest survey revealed that the most affected patients were female housewives (40.9%) and students (18.18%) in Herat Province, Afghanistan, with the main route of transmission being the consumption of contaminated dairy products [22]. Brucellosis was introduced in Sri Lanka during the Second World War through the importation of cattle [23]. A seroprevalence of 8.4% (109/1,294) of human *Brucella* infection was reported, with *B. abortus* the most common species detected serologically. Farm animal owners and those working full-time with livestock have a significantly higher risk of acquiring *Brucella* infection [24]. The disease is common and endemic in humans in Pakistan. It has been reported that among all provinces, the highest numbers of patients were from Punjab and Khyber Pakhtunkhwa Provinces, but the disease is still considered highly underreported and misdiagnosed [25]. The seroprevalence of brucellosis in humans was 2.0%–70% from 2000 to 2020 [26].

Although brucellosis has been reported to be endemic in Nepal, there is no available data from recent decades. Previous reports showed that the overall prevalence of brucellosis was 100 cases/10,000 inhabitants, which decreased to less than 20 cases/10,000 in 2003 [27]. Brucellosis is a significant public health concern in India because of its high seroprevalence in humans; however, epidemiological data are lacking. A recent survey showed that the overall seroprevalence of brucellosis was 11.37% in Meghalaya [28], 29.73% in Sambalpur District, and 4.44% in Mayurbhanj District [29]. The annual median losses due to human brucellosis were estimated to be Rs 627.5 million among adults and Rs 185.0 million among children [30].

In Western Asia, brucellosis is highly endemic in humans in the majority of countries. In Iran, the incidence rate of human brucellosis continuously declined from 38.06/100,000 in 2005 to 11.40/100,000 in 2012 and subsequently increased to 20.33/100,000 in 2018 (Figure 3b). Although the incidence of human brucellosis has declined since 2015, the number of cases has remained high, with 138,448 cases of brucellosis reported from 2009 to 2017 at an annual incidence rate of 21.60/100,000 [31]. Human brucellosis was epidemic in Turkey, originally showing a decreasing incidence rate that subsequently increased. The incidence rate was 21.57/100,000 in 2005, declined to 5.31/ 100,000 in 2015, and then sharply increased to 12.28/100,000 in 2019 (Figure 3b). Adherence to traditional farming practices and lifestyles, and the consumption of fresh dairy products contribute to the high incidence of brucellosis. The incidence of human brucellosis in 2005 of 3.31/100,000 increased to 5.05/ 100,000 in 2019 in Georgia, with a peak incidence in 2014 of 7.58/100,000. Similarly, brucellosis in Jordan has shown an increasing trend. A survey found that 31.1% (456/1,497) of febrile disease cases were brucellosis, and seropositivity was highest among dairy factory workers at 64.4% [32]. Historically, Syria has experienced the highest human brucellosis burden. A total of 137,609 cases were reported between 2007 and 2019; however, the incidence rate in 2007 (200.41/100,000) had declined to 36.64 /100,000 by 2019 (Figure 3b). In Armenia, the incidence rate of human brucellosis has fluctuated, with an increase in 2007 from 9.94/100,000 to 13.08/100,000 in 2014, and then gradually decreasing to 8.32/100,000 in 2019 (Figure 3b).

Brucellosis is still an endemic disease in Lebanon, with increases in cases primarily in the southern and northern areas [33]. A brucellosis outbreak in the Chouf district was reported in 2009, in which consumption of raw cheese was a significant risk factor [34]. The Lebanese Ministry of Public Health reported that Bekaa District had the highest human brucellosis incidence over the years, with 300 cases/1,000,000 individuals, followed by Mount Lebanon and South Lebanon [35]. Notably, a similar re-emerging pattern was reported in the neighbouring Israeli regions. In Israel, three distinct periods of large outbreaks of *B. melitensis* in humans were documented since 1951. The first outbreak period was 1950–1960, after which cases decreased by the early 1960s. The second outbreak period began around 1984 and lasted for about 15 years, and the third outbreak occurred from 2013 to 2020 and peaked in 2014 [36]. The incidence of human brucellosis in the general population in Israel increased sharply from 1.9/100,000 in 2009 to a peak of 7.3/100,000 in 2014, and eventually decreased to 3.0/100,000 in 2019. In Palestine, there was a dramatic and continuous increase in human cases, from 3.79/100,000 in 2005 to 15.32/100,000 in 2018, reaching a peak in 2016, with an incidence of 27.18/100,000 (Figure 3b). The re-emergence of brucellosis was due to *B. melitensis* strains of local origin. The weakness of the Palestinian and Israeli control programs may have been a major factor behind the re-emergence of the disease [37]. Brucellosis is a common zoonotic disease of major concern in humans in Kuwait, and *B. melitensis* causes most of the human cases [38]. The incidence rate in 2005 increased from 2.64/100,000 to 12.25/100,000 in 2018 and then decreased to 9.82/100,000 in 2019. These data indicate that strengthened surveillance is needed to understand the epidemiological situation.

Human brucellosis is an ongoing epidemic in Qatar, Saudi Arabia, Azerbaijan, Oman, and Yemen because of the traditional feeding management systems for animals practiced in these countries, in which animal owners are frequently directly involved in livestock care and management. The incidence of brucellosis in Qatar was lower than that in other Western Asian countries; however, the number of cases increased from 26 (3.0/100,000) in 2005 to 132 (4.3/100,000) in 2019 (Figure 3c). One of the main risk factors is the cultural acceptability of raw, unpasteurized camel milk [39].

Between 2004 and 2012 in Saudi Arabia, there were 37,477 reported cases of human brucellosis [40]. The number of reported cases was 3,233, 4,062, and 4,692 in 2015, 2016, and 2017, respectively, and the incidence rate increased in this period from 10.19/100,000 in 2015 to 14.17/100,000 in 2017. These data reveal that Saudi Arabia has a high disease burden; however, intermittent surveillance does not depict the true epidemiological profile. In Azerbaijan, the disease has appeared to increase and then decrease in an alternating pattern, in which the incidence of 5.06/100,000 in 2005 decreased to 1.70/100,000 in 2009, then increased to 4.64/100,000 in 2011, decreased again to 0.70/100,000 in 2014, and then continuously increased to 5.58/100,000 in 2019 (Figure 3c). Moreover, the disease appears to be re-emerging in southeastern areas, although there are decreases in cases elsewhere in the country [41]. Although brucellosis occurs frequently in Yemen, its epidemiology has not been extensively studied. The incidence rate reported in 2015, 2016, and 2018 was 46.62/100,000, 92.17/100,000, and 15.76/100,000, respectively. However, epidemic situation of the disease has suffered from being underreported, and the actual epidemic status needs to be determined with further surveys [42]. Brucellosis is the most common zoonotic disease in Oman. The incidence rate showed an upward trend from 4.5/100,000 in 2005 to 9.3/100,000 in 2016, and then declined to 6.3/100,000 in 2018 (Figure 3d). These data reveal that surveillance and control of human brucellosis in these countries are extremely difficult due to the traditional lifestyle, poor economic and health conditions, the lack of adequate control programs in some of these countries, uncontrolled animal transportation through “open borders,” all of which increase the risk that brucellosis will spread in these high disease burden regions. Interventions implemented in these regions over decades have led to a decrease in human cases. However, each period of low incidence rates over a few years was followed by a subsequent increase in cases. The present analysis highlights that human brucellosis has become an insuperable social-economic concern in these countries. Continuous financial investments, tailored public policies, and the active mobilization of social resources are indispensable for curbing the spread.

## Europe

Based on the Annual Epidemiological Reports of the European Centre for Disease Prevention and Control (an agency of the European Union), major European countries are considered to have a brucellosis-free status or a low prevalence of human brucellosis (Figure 1). In the EU/EEA, the annual total number of reported cases ranged between 358 and 1,349, and the notification rate was between 0.08 and 0.2/100,000 for the 2005–2020 period. However, despite the incidence gradually declining annually, brucellosis is persistently reported in several previously high disease-burden countries of Southern and Central Europe, including Greece, Albania, North Macedonia, Bosnia and Herzegovina, Italy, Spain, and Portugal. Moreover, sporadic and imported cases were reported in some low-burden countries, such as France, Germany, and the United Kingdom.

Human brucellosis has been recorded in all regions of Greece; however, the incidence rate has gradually decreased from 3.01 (*n*=331) in 2005 to 0.28 (*n*=65) in 2020, with a slightly increasing trend from 2012 to 2014 (Figure 4a). In Portugal, Spain, the Russian Federation, and Italy, the incidence rate fluctuated but generally declined from 2005 to 2019 (Figure 4a). In Portugal, brucellosis is a notifiable disease, and human cases are reported in all regions of continental Portugal. The incidence in Portugal decreased from 1.62 in 2005/100,000 to 0.155/100,000 in 2017, with a slight increase to 0.33/100,000 in 2019 (Figure 4a). In Spain, the incidence rate was 0.67/100,000 person-years from 1997 to 2015 with a progressive decrease in the number of cases and annual incidence rates. The reported cases declined from 328 cases in 2005 to 25 cases in 2021. In Italy, the incidence of human brucellosis decreased from 2.7 /100,000 in 1990 to 1.4 /100,000 in 2002, followed by a persistent decrease to 0.03/100,000 in 2020. The disease is widespread mainly in the southern regions of Italy, especially in Sicily [43]. In Russia, the incidence of human brucellosis was stable around 0.3/100,000 from 2005 to 2019, but with a slightly increasing trend recently (Figure 4a). However, in the North Caucasian Federal District, the largest number of new human brucellosis cases were reported in the Republic of Dagestan (59.3%) and Stavropol Territory (27.4%) from 2000 to 2014, with the incidence of brucellosis in the Stavropol Territory 5–10-fold higher than that in the Russian Federation as a whole [44]. Thereafter, continued strengthening of surveillance and implementation of control policies in these areas became necessary.

Brucellosis has been endemic in Albania for a long time. DNA sequencing of bone lesions of two adolescent males from the 10th to 13th centuries AD confirmed that brucellosis was endemic to the area since at least the Middle Ages [45]. During the socialist era, brucellosis was virtually eradicated in humans in Albania, with only three human cases recorded in 1985. However, between 1994 and 2004, human cases increased roughly 10-fold, with a total of 1,149 confirmed cases in 2004. The overall reduction in the number of human cases has been attributable to the effects of vaccination [46]. Subsequently, the number of cases slightly increased from 700 cases (23.2/100,000) in 2005 to 932 (31.3/100,000) in 2007, and then declined from 790 cases (26.8/100,000) in 2008 to 35 (1.2/100,000) cases in 2018 (Figure 4b). However, surveillance of the evolution of the disease is necessary to assess the risk factors in a timely manner.

Human brucellosis is an emerging infectious disease in Bosnia and Herzegovina. Owing to the implementation of eradication measures in the 1980s and the 1990s, Bosnia and Herzegovina were free of brucellosis from 1980 to 2000 [47]. After this time, brucellosis cases rapidly increased, and infections have been recorded in almost the entire territory. The total number of human brucellosis cases reported in the period 2001–2008 in the country was 1,708, and 2,090 cases were reported from 2009 to 2019. The incidence from 3.64/100,000 in 2005 increased to 27.01/100,000 in 2008, after which there was a consistent decline to a low in 2012 (1.89), followed by an increase from 2.48/100,000 in 2013 to 10.77/100,000 in 2018, and a subsequent decrease to 6.24/100,000 in 2019 (Figure 4b). Brucellosis has been present continuously with a varying morbidity rate and an overall increasing incidence rate; therefore, implementing a comprehensive surveillance strategy is recommended to counter the epidemic situation.

Brucellosis is an endemic disease in northern Macedonia. From 1980 to 2009, a total of 11,451 brucellosis cases were reported, with a mean annual incidence rate of 18.9/100,000, with the highest morbidity rate during this period recorded in 1992 (922 cases and an incidence rate of 47.6/100,000), and the lowest in 1983 (12 cases and an incidence rate of 0.6/100,000) [48]. There was a low epidemic level (8.5%, 34/398) from 1998 to 2009, which was evidenced by the ongoing circulation of brucellosis [49]. Subsequently, the incidence dramatically increased and peaked in 2008 (23.27, *n* = 481), after which the human incidence rate gradually declined from 13.87/100,000 in 2009 to 0.72/100,000 in 2019 (Figure 4b). Generally, the incidence of human brucellosis in Europe has gradually declined; however, because of intrinsic husbandry practices, traditional food, and the living habits in some countries, regular surveillance and control countermeasures are needed to better depict and control the pattern of brucellosis infections in this region.

## Oceania

Oceania is a human brucellosis-free continent, with only sporadic cases reported in Australia and New Zealand. A total of 398 cases were reported between 2005 and 2018, and an average of 28 cases were diagnosed annually in Australia. Currently, *B. suis* is the only cause of endemic brucellosis in Queensland due to human killing and handling of feral pig carcasses. Other human cases of brucellosis were imported from endemic countries such as Mediterranean, Middle East, and Latin American countries [50]. In addition, only 19 human cases were reported in New Zealand between 2007 and 2019. However, owing to frequent international trade, animal movement, and immigration, its incidence is increasing, and continuous surveillance is needed in non-endemic areas to evaluate any changes.

## Africa

Human brucellosis is a severe disease that affects Africa. There is a significant lack of available data owing to weak public health surveillance systems and low socioeconomic development; therefore, it is difficult to understand the actual incidence of the disease. Although human brucellosis is known or suspected to exist in 40 of the 49 African countries (82%), in 20 (41%) of these it represents a major problem for human health and the economy [51]. More than 96% of countries in Africa do not have continuous surveillance or report the status of human brucellosis. However, published data related to infection events and serological and molecular detection of human brucellosis has shown a gradual increase in the disease.

Human brucellosis is endemic in Northern Africa, where it was first identified in 1895. In Algeria, the most affected region is the steppe region, which alone has an average incidence (cases/100,000 inhabitants) of 65.87, followed by 9.89 in the north [52]. A total of 91,985 human cases were reported from 2005 to 2016 and 2018, with a peak in 2018 of 11,423 cases. The incidence rate fluctuated with an overall decrease from 25.6/100,000 in 2005 to 10.9/100,000 in 2013, then increased from 15.7/100,000 in 2014 to 27.1/100,000 in 2018. In addition, 13,670 human brucellosis cases were reported in Tebessa Province from 2000 to 2020, with the annual notification rate ranging from 30.9/100,000 (2013) to 246.7/100,000 (2005) [53]. In Egypt, a total seroprevalence of brucellosis in humans ranged between 5% to 8%, with no significant differences with respect to different seasons of the year [54]. In Tunisia, the incidence of human brucellosis increased from 2.81/100,000 in 2005 to 8.62/100,000 in 2018, during which time the number of cases increased from 284 (2005) to 997 (2018). In addition, an outbreak of 25 cases was reported in Douz, Tunisia in 2018, and consumption of raw milk from smuggled sick goats was the main risk factor in this outbreak [55]. In Libya, human samples obtained from 2006 to 2008 showed a high seropositivity of 40%, with 95 (43%) of the 221 positive samples positive for IgM, indicating an active or recent infection [56]. In Morocco, the seroprevalence of brucellosis was 33.20% (426/1,283) with a higher risk among males and rural residents, suggesting that a very large proportion of the population in this region was already infected with *Brucella* [57]. These data demonstrate that human brucellosis poses a serious challenge to the health of humans, with rampant outbreaks and spillover risks.

In West Africa, a comprehensive phylogenetic analysis of the pathogen *Brucella abortus* revealed the consistent presence of *B. abortus* across West Africa and its neighbouring countries. The study also concluded that the majority of local cases in West Africa were caused by native strains, while some of the cases could be traced back to introduced lineages [58]. A serum epidemiological study from 1982 demonstrated the presence of human brucellosis in all regions in Mali [59]. A serology survey showed that human brucellosis existed in Benin, and the percentage of positive serum samples among exposed workers was 17.7% [60]. The human seroprevalence for *Brucella* spp. was 5.3% (88) in northern Côte d'Ivoire between 2012 and 2014 [61]. Human brucellosis is endemic in Nigeria, where it lacks appropriate attention and national data. A systematic review showed that the national seroprevalence of human brucellosis was 17.6% (554/3,144) from 2001 to 2021, and the peak was observed in 2016 in southeastern Nigeria [62].

Although there was very limited surveillance data on human brucellosis in Central Africa, it has been reported in some of the countries. The overall prevalence of human brucellosis was estimated at 0.2% in two rural health districts in Chad [63]. The human *Brucella* seroprevalence was 5.6% among abattoir personnel and 0.28% in pregnant women in Ngaoundéré, Cameroon [64], with the presence of multiple *Brucella* spp. [65]. These data suggest the presence of human brucellosis in the area, and attention and enhanced surveillance are warranted to understand the epidemic status.

In East African countries, the reported prevalence of human brucellosis among patients at hospitals and exposed pastoral and agro-pastoral communities varied from 0.0% to 35.8% from 2010 to 2019 [66]. The seropositivity of patient blood samples for brucellosis was 23.3% (97/416) among patients presenting to Wau Hospital in South Sudan [67]. In Kenya, bacteriological evidence revealed the presence of *B. abortus* and *B. melitensis* in human patients [68]. The reported cases increased from 84,755 in 2013 to 154,096 in 2019, corresponding to an incidence of 186.24/100,000 in 2013 and 293.10/100,000 in 2019. In Eritrea, the incidence continuously increased from 0.33/100,000 in 2014 to 44.11/100,000 in 2018, and the number of cases jumped from 11 in 2014 to 1,523 in 2018. These data reveal that human brucellosis has been rampant epidemic in these countries, and regular surveillance and control strategies are highly recommended. Human brucellosis in Baringo was mainly caused by *B. abortus*, in which 26.5% of cases were positive using Febrile Brucella Antigen agglutination test and 10.2% were positive using Rose Bengal Plate Test [69]. Brucellosis is highly prevalent in the Kiboga District, Uganda, and the transmission risk was aggravated by the consumption of unpasteurized milk products and residing in rural settings [70]. In Ethiopia, the pooled seroprevalence of brucellosis in humans was 5% from 2000 to 2020, during which time the public awareness of brucellosis was low (18.4%), while practices that exposed humans to *Brucella* infections were high [71].

In South Africa, brucellosis caused by *B. abortus* has been an important medical condition for more than a century. However, it is underdiagnosed and underreported because many clinicians have little or no experience in managing affected patients, in addition to the nonspecific and insidious nature of the disease [72]. The serological prevalence of human brucellosis in Namibia was 11.64% (113/971) from 2012 to 2017 [73]. Human brucellosis is endemic in northern Tanzania, with an incidence of 35 cases/100,000 persons annually in 2007 and 2008, and 33 cases /100,000 persons annually in the period 2012–2014 [74]. These data sufficiently demonstrate that human brucellosis is an endemic and persistently epidemic disease in Africa, and that little is known about the epidemiology of human brucellosis in this continent, hindering surveillance and control efforts at the individual and population levels. Therefore, increasing attention to the disease and building surveillance systems is a priority for implementation in targeted populations to better understand the epidemiological trend.

## America

Human brucellosis a reemerging disease in the Americas. Human brucellosis cases have been reported with varying prevalence in all American countries, including North and Latin America. However, most of these cases have been recorded in Latin America, including Mexico, Peru, Argentina, the United States, and Brazil [75]. The epidemic situation in other countries is relatively poor, with an average annual number of cases of less than 30.

**Figure 5.**
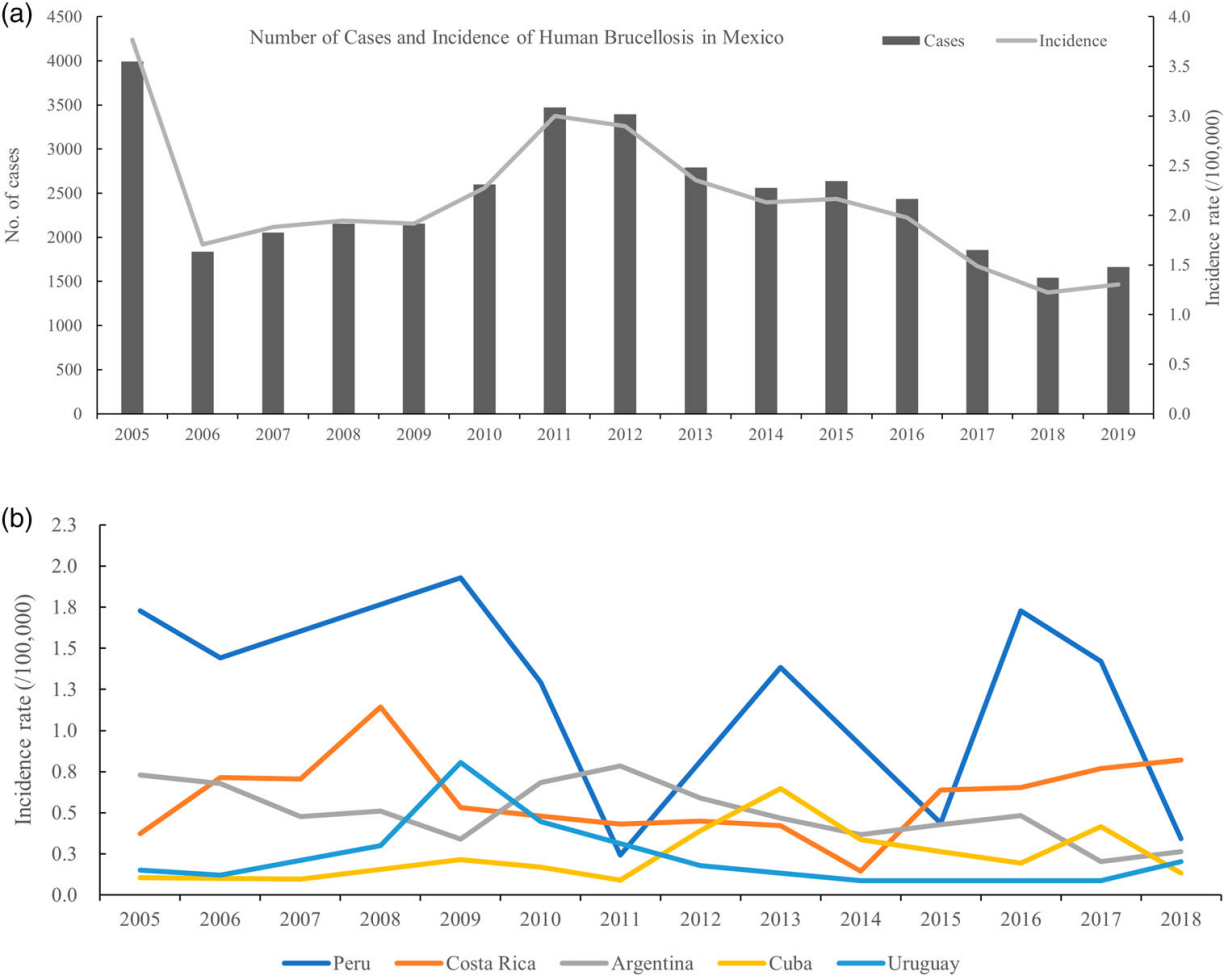
The incidence rate (/100,000) profile in American countries (a (Mexico) and b) with a high disease burden, from 2005 to 2019. Note: the number of cases and the incidence rates of human brucellosis are from public open-source from OIE-WAHIS databases.

Mexico is still a hotspot area and has the highest disease burden in Latin America. The number of cases from 1990 to 1993 sharply increased, and the number of human brucellosis recorded cases was 5,620, 5,788, 5,958 and 5,134 cases in the four years, respectively [76]. This was followed by a declining trend in cases from 1994 (n = 3,518) to 2001 (n = 2,107), and then a slightly increased trend from 2002 to 2005. From 2005 to 2019, a total of 37,157 cases were reported and the average number of reported cases annually was 2,477. The incidence showed an overall decrease from 3.77/100,000 in 2005 to 1.30/100,000 in 2019 (Figure 5a), but it still suggested a high brucellosis burden. The high disease burden was mainly associated with the consumption of unpasteurized dairy products (fresh cheese) and close contact with cattle [76].

Although human brucellosis in Peru is historically endemic, the absence of a national policy or differential diagnostic tests hinders the development of surveillance and control programs in high-risk rural areas [77]. From 2005 to 2019, a total of 3,523 cases were intermittently reported, and the average number of reported cases annually was 352. The reported peak occurred in 2009 (*n* = 555, 1.93/100,000), followed by a decrease in 2016 (*n* = 534, 1.73/100,000), with the lowest cases in 2011 (*n* = 71, 0.24/100,000) (Figure 5b). It is necessary to develop regular surveillance programs to better assess the epidemic changes in a high-risk area.

In Argentina, the first human brucellosis was described in 1922, and *B. melitensis* was first isolated from a patient in 1930. Subsequently, approximately 1,000 *Brucella* strains were isolated from humans in Latin American countries between 1968 and 1991, and 367 strains were isolated from humans in Argentina from 1994 to 2006 [75]. Isolation of *Brucella* spp. from humans provides irrefutable evidence of the infection and its endemic status. A total of 3,120 cases were reported from 2005 to 2019, during which time the incidence rate fluctuated with an overall decline from 0.73/100,000 in 2005 to 0.21/100,000 in 2017, followed by a slightly increasing trend in 2018 (0.26/100,000) and 2019 (0.56/100,000) (Figure 5b). *B. melitensis* remains the principal cause of infection, but surveillance and control of human brucellosis is a considerable challenge, and the construction of a nationally coordinated diagnostic net would contribute to better controlling the disease in humans.

In the United States, brucellosis remains a disease risk to people through the acquisition and consumption of animal products from endemic countries, such as Mexico. There has been a significant decrease in cases observed in the USA, with 6,321 cases reported in 1947 and only 139 cases reported in 2018. From 1993 to 2017, however, 492 cases of brucellosis were reported in California residents, and *B. melitensis* was identified in 80% (393/492) of patients, with more than 34% (168/492) specifically reporting consuming unpasteurized dairy products from Mexico [78]. Therefore, targeted public health measures are needed to prevent the potential risk to populations in highly burdened regions, such as California and Texas, including implementing strict border control and advocating the consumption of pasteurized dairy products.

Since the first case was reported in 1934 in Brazil, cases have been reported throughout the country. *B. abortus* is the most prevalent species in Brazil, and cases in humans were all caused by *B. abortus* but had not been shown to be caused by *B. melitensis.* Human brucellosis cases increased from 2009 to 2019, during which the incidence increased from 0.01/100,000 in 2009 to 0.11/100,000 in 2019 (Table S1). In addition, human brucellosis has been intermittently reported in many countries (Figure 5b), including Costa Rica, Cuba, Uruguay, Venezuela (Bolivarian Republic of), and Ecuador from 2005 to 2018, during which the total reported cases were 385, 345, 87, 191 and 136, respectively. In particular, 45 cases (0.26/100,000) were reported in Ecuador in 2019, with an ongoing increasing trend observed in recent years [80]. Overall, strengthening the surveillance of human brucellosis in these countries is recommended to facilitate an understanding of the epidemic evolution and to develop a control plan.

Although this study offers new insights, it has certain limitations. First, the present analysis may only partially reveal the true epidemic situation owing to widespread information deficiency. Second, there are differences in case definitions, test assays, and reagents in each country, and to a certain extent these may lead to bias in the results. Finally, an epidemic analysis conducted on animal brucellosis supported the conclusions of this study.

## Conclusions

Despite improvements in its diagnostic and surveillance capacity, brucellosis remains a serious global public health burden. The epidemiological characteristics of human brucellosis have changed notably over the past few decades and are driven by multiple social factors. Human brucellosis in areas where it is traditionally endemic, such as the Mediterranean and surrounding countries, is persistently circulating, and re-emergence is frequently reported in completely controlled countries in North America and South Europe. Therefore, implementing surveillance and testing is crucial for responding in a timely manner to these changes, especially in low-income countries such as those in Asia. For example, the detection and surveillance of brucellosis and the use of new technologies must be significantly enhanced. Additionally, the concepts of prevention, sources, and surveillance technologies are extremely limited to low-income countries. In particular, little attention has been paid to Africa; however, brucellosis is historically endemic to Africa. There are major gaps in epidemiological surveillance and the understanding of zoonotic concepts surrounding brucellosis on the African continent. Decreasing the burden of human brucellosis requires the control of animal brucellosis, and evidence to inform the design of control programs in the developing world is needed. Brucellosis knows no boundaries, as the epidemiology of this zoonosis changes with changing lifestyles and evolving human-animal interaction. “One health” is a vital concept for an effective investigation into the human–animal–ecosystem interface for prevention and control of brucellosis re-emergence.

## Supplementary Material

Table_S1

Certificate_of_english_editing
