# Supplementary material for: Long ignored but making a comeback: a worldwide epidemiological evolution of human brucellosis
Source: Emerg Microbes Infect. 2023 Dec 1;13(1):2290839. doi: 10.1080/22221751.2023.2290839 (PMC10878345; doi:10.1080/22221751.2023.2290839)
Supplement: Certificate_of_english_editing [file TEMI_A_2290839_SM9066.pdf]

CERTIFICATE OF ENGLISH EDITING

This document certifies that the paper listed below has been edited to ensure that the language is clear and free of errors. The logical presentation of ideas and the structure of the paper were also checked during the editing process. The edit was performed by professional editors at Editage, a division of Cactus Communications, in cooperation with Taylor & Francis Group. The intent of the author's message was not altered in any way during the editing process. The quality of the edit has been guaranteed, with the assumption that our suggested changes have been accepted and have not been further altered without the knowledge of our editors.

Title

Long ignored but making a comeback: a worldwide epidemiological evolution of human brucellosis

Authors

Zhiguo Liu

Order No.

URJHY_1

EDITINGSERVICES
Supporting Taylor & Francis authors

Signature

Prabh Grewal,
Senior Vice President,
Editage

Date of Issue
November 25, 2023
